# Contrast-Enhanced Ultrasound (CEUS) for the Evaluation of Bosniak III Complex Renal Cystic Lesions—A 10-Year Specialized European Single-Center Experience with Histopathological Validation

**DOI:** 10.3390/medicina56120692

**Published:** 2020-12-12

**Authors:** Vincent Schwarze, Johannes Rübenthaler, Saša Čečatka, Constantin Marschner, Matthias Frank Froelich, Bastian Oliver Sabel, Michael Staehler, Thomas Knösel, Thomas Geyer, Dirk-André Clevert

**Affiliations:** 1 Department of Radiology, University Hospital LMU, Marchioninistrasse 15, 81377 Munich, Germany; vincent.schwarze@med.uni-muenchen.de (V.S.); johannes.ruebenthaler@med.uni-muenchen.de (J.R.); sasa.cecatka@googlemail.com (S.Č.); constantin.marschner@med.uni-muenchen.de (C.M.); bastian.sabel@med.uni-muenchen.de (B.O.S.); dirk.clevert@med.uni-muenchen.de (D.-A.C.); 2 Department of Radiology and Nuclear Medicine, University Medical Centre Mannheim, Theodor-Kutzer-Ufer 1-3, 68167 Mannheim, Germany; Matthias.froelich@medma.uni-heidelberg.de; 3 Department of Urology, University Hospital LMU, Marchioninistrasse 15, 81377 Munich, Germany; Michael.Staehler@med.uni-muenchen.de; 4 Institute of Pathology, University Hospital LMU, Marchioninistrasse 15, 81377 Munich, Germany; Thomas.Knoesel@med.uni-muenchen.de

**Keywords:** contrast-enhanced ultrasound, CEUS, Bosniak III, renal cysts, RCC

## Abstract

*Background and objectives*: The aim of the present retrospective single-center study is to evaluate the diagnostic performance of contrast-enhanced ultrasound (CEUS) for assessing Bosniak III complex renal cystic lesions with histopathological validation. *Materials and Methods*: 49 patients with CEUS-categorized Bosniak III renal cystic lesions were included in this retrospective study. All patients underwent native B-mode, Color Doppler, contrast-enhanced ultrasound (CEUS) between 2010–2020. Eight and five patients underwent computed tomography (CT) and magnetic resonance imaging (MRI), respectively. Twenty-nine underwent (partial) nephrectomy allowing for histopathological analysis. The applied contrast agent for CEUS was a second-generation blood pool agent. Ultrasonography examinations were performed and interpreted by a single experienced radiologist with more than 15 years of experience (EFSUMB Level 3). *Results*: CEUS examinations were successfully performed in all included patients without registering any adverse effects. The malignancy rate of CEUS-categorized Bosniak III renal lesions accounted for 66%. Initially, cystic complexity was visualized in native B-mode. In none of the renal lesions hypervascularization was detected in Color Doppler. CEUS allowed for detection of contrast enhancement patterns in all included Bosniak III renal lesions. Delayed wash-out could be detected in 6/29 renal lesions. In two cases of histopathologically confirmed clear-cell RCC, appropriate up-grading from Bosniak IIF to III was achieved by CEUS. *Conclusions*: CEUS depicts a promising imaging modality for the precise diagnostic workup and stratification of renal cystic lesions according to the Bosniak classification system, thereby helping guidance of adequate clinical management in the future.

## 1. Introduction

Renal cell carcinoma (RCC) accounts for up to 3% of all cancer entities, 30% of patients with RCC present with metastatic disease [1]. It represents the urological cancer with the highest mortality rates of up to 40%. The detection rates of RCC have significantly increased during the last decades mainly owing to advancing imaging technologies. Still, a relevant proportion of incidentally found renal lesions remains indeterminate and necessitates further diagnostic. Up to 8% of RCCs appear as complex cystic lesions.

Cystic renal lesions appear in over 50% of people of 50 years of age or older [2]. Due to the increasing use of more advanced cross-sectional imaging, mainly computed tomography (CT) and magnetic resonance imaging (MRI), incidental renal lesions are more frequently detected, the majority of which reveals to be simple and uncomplicated cysts [3]. Nevertheless, incidentally found renal lesions may feature complicating characteristics in up to 10%, encompassing contrast enhancement, intracystic septa, mural thickening, calcifications or nodular components, thus remaining indeterminate and may show malignant potential. Therefore, close follow-up examinations and/or surgery and histopathologic elucidation are indispensable.

Since its introduction in 1986, the Bosniak classification system has helped radiologists to classify renal lesions and helped estimate the likeliness of benign and malignant origin based on CT criteria [4]. Upon later modification, renal cysts can be subdivided into five different Bosniak subtypes (I-IV and IIF, “F” = follow-up). Whereas Bosniak I and II subtypes have a malignancy rate of nearly 0%, IIF, III and IV are associated with malignancy rates of approximately 5%, 50% and 100% respectively [5,6]. The morphologic boundaries between benign and malignant renal lesions often are blurred. Of note, classifying renal lesions to a Bosniak subtype determines therapeutic management of the patient. It is therefore obvious that a precise assessment and classification of renal lesions is pivotal in order to guide adequate clinical management of the patient. The latest proposed update of the Bosniak classification system was published in 2019 [7]. Of note, it incorporates MRI features of renal lesions into the stratification. Besides CT and MRI and its proven accurate diagnostic performance for renal imaging, contrast-enhanced ultrasound (CEUS) is still not included in the latest Bosniak classification system.

Contrast enhancement is a critical morphologic feature which may allow to distinguish between malignant and benign origin of the renal lesion. It can be detected by using elaborate CT or MRI. Both imaging modalities need thorough evaluation before being performed due to ionizing radiation in case of CT, potential allergic reactions to iodinated or gadolinium-based contrast agents, potential renal and thyroid gland impairments or possible metallic medical devices, e.g., cardiac pacemakers in patients. Conventional ultrasound, including native B-mode and Color Doppler, is not feasible to visualize contrast enhancement of renal lesions. In contrast and as its name implies, CEUS overcomes the shortcomings of conventional ultrasound and allows for dynamic visualization of microperfusion [8] at higher spatial and temporal resolutions compared to CT and MRI and has previously already proven high diagnostic accuracy in renal imaging, in particular in the differentiation of renal masses and clarifying indeterminate renal lesions [9,10]. CEUS depicts a strikingly sensitive imaging tool by which even single microbubbles can be visualized within a cystic wall or septum [11]. It may therefore assist with precise stratification of renal lesions by appropriate categorization into Bosniak subtypes [12]. Noteworthy, CEUS is inexpensive, easily accessible and repeatable and has an excellent safety profile [13]. The advantageous role of CEUS for visualization of complex renal lesions was already described in several clinical studies [14,15,16,17]. It could be shown that CEUS allows for equivalent diagnostic performance compared to CT and MRI for assessing complex renal lesions.

The aim of the present European single-center study is to evaluate the diagnostic performance of CEUS in comparison with histopathology for assessing Bosniak III renal cystic lesions.

## 2. Materials and Methods

This retrospective single-center study was approved by the local institutional ethical committee of the institutional review board (Date of Approval: 14 March 2017, Ethic Code: 17-087) and all contributing authors followed the ethical guidelines for publication in *Medicina.* All study data were gathered according to the principles expressed in the Declaration of Helsinki/Edinburgh 2002. Oral and written informed consent of all patients were given prior to each CEUS examination and their associated risks and potential complications have been carefully described. All CEUS examinations were performed and analyzed by a single skilled radiologist with more than 15 years of clinical experience in advanced ultrasound techniques (EFSUMB Level 3). All included patients underwent native B-mode, Color Doppler and CEUS scans. At the time of the examination, up-to-date high-end ultrasound systems with adequate CEUS protocols were utilized (Siemens Ultrasound Sequoia S2000, S3000, Siemens, Mountain View, CA, USA; Philips Ultrasound iU22, EPIQ7, Philips, Seattle, WA, USA). A low mechanical index was used in all cases to avoid early destruction of microbubbles (<0.2). For all CEUS examinations, the second-generation blood pool contrast agent *SonoVue*^®^ (Bracco, Milan, Italy) was used. Then, 1.0–2.4 mL of *SonoVue*^®^ was applied. After contrast agent was applied, a bolus of 5–10 mL sterile 0.9% sodium chloride solution was given. No adverse side effects upon administration of *SonoVue^®^* could be observed. All CEUS examinations were successfully performed and image quality was sufficient in all cases allowing for proper diagnostic analysis of the sonomorphological appearance of the renal lesions. The patient files and imaging records were retrieved from the picture archiving and communication system (PACS) of our institution for further analysis.

The vascular phases of CEUS comprised cortical phase (8–35 s after i.v. application), corticomedullary phase (36–120 s after i.v. application) and late phase (>120 s to the disappearance of the microbubbles). Dynamic contrast differences in the perfusion of the renal parenchyma compared to the lesions were evaluated with qualitative analysis of wash-in and wash-out characteristics. Evaluation of morphological features included: location, size, shape and echogenicity of the lesions. Vascularization was assessed using Color Doppler and CEUS. Retrospective analysis of archived cine-loops of all included patients was performed.

**Figure 1:**
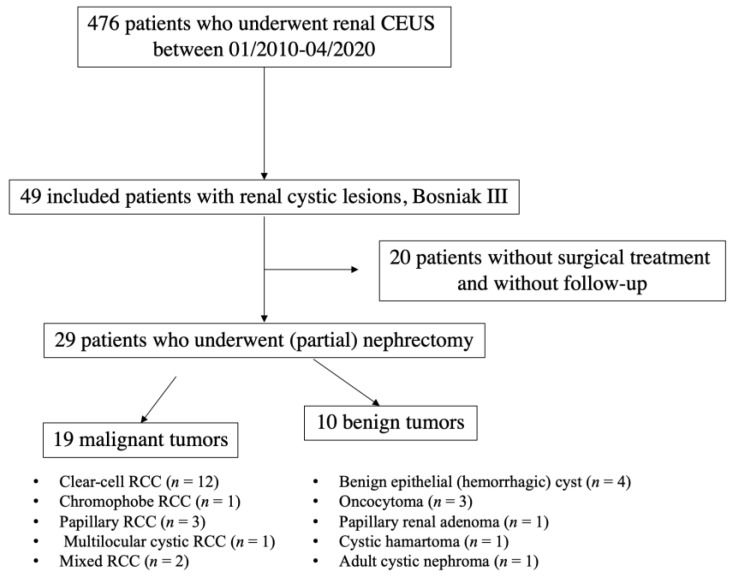
Flowchart illustrating selection of patients with Bosniak III renal cystic lesions. CEUS: contrast-enhanced ultrasound; RCC: renal cell carcinoma.

Between 01/2010–04/2020, 476 patients in total underwent renal contrast-enhanced ultrasound (Figure 1). Forty-nine patients with renal lesions categorized as Bosniak type III by CEUS were included in this retrospective single-center study. Eight patients additionally underwent CT scan; five patients underwent additional MRI.

Twenty-nine of 49 patients underwent (partial) nephrectomy in the local Department of Urology. The histopathological analysis was performed in collaboration with the local Institute of Pathology. Histopathological results were used as the diagnostic reference standard. 

## 3. Results

CEUS examinations were performed in all 49 included patients without registration of any adverse effects (Supplementary Table S1). The female to male ratio was 1: 2.2. In total, 26 renal lesions were located on the left side, 23 renal lesions were located on the right side (left: right–ratio = 1.1: 1). The average diameter of the renal lesions was 3.4 cm (min = 0.8 cm, max = 12.0 cm). The mean age of the patient at the time of CEUS examination was 64 years (range: 35–92 years). Histopathological analysis revealed the following renal cell carcinoma (RCC) subtypes listed with decreasing frequencies: 41% (12/29) clear-cell RCC, 10% (3/29) papillary RCC, 7% (2/29) mixed RCC, 3% (1/29) multilocular cystic RCC and 3% (1/29) chromophobe RCC; benign entities comprised 14% (4/29) benign epithelial cysts, 10% (3/29) oncocytoma and 3% (1/29) papillary renal adenoma, cystic hamartoma and adult cystic nephroma (Table 1).

**Figure 2:**
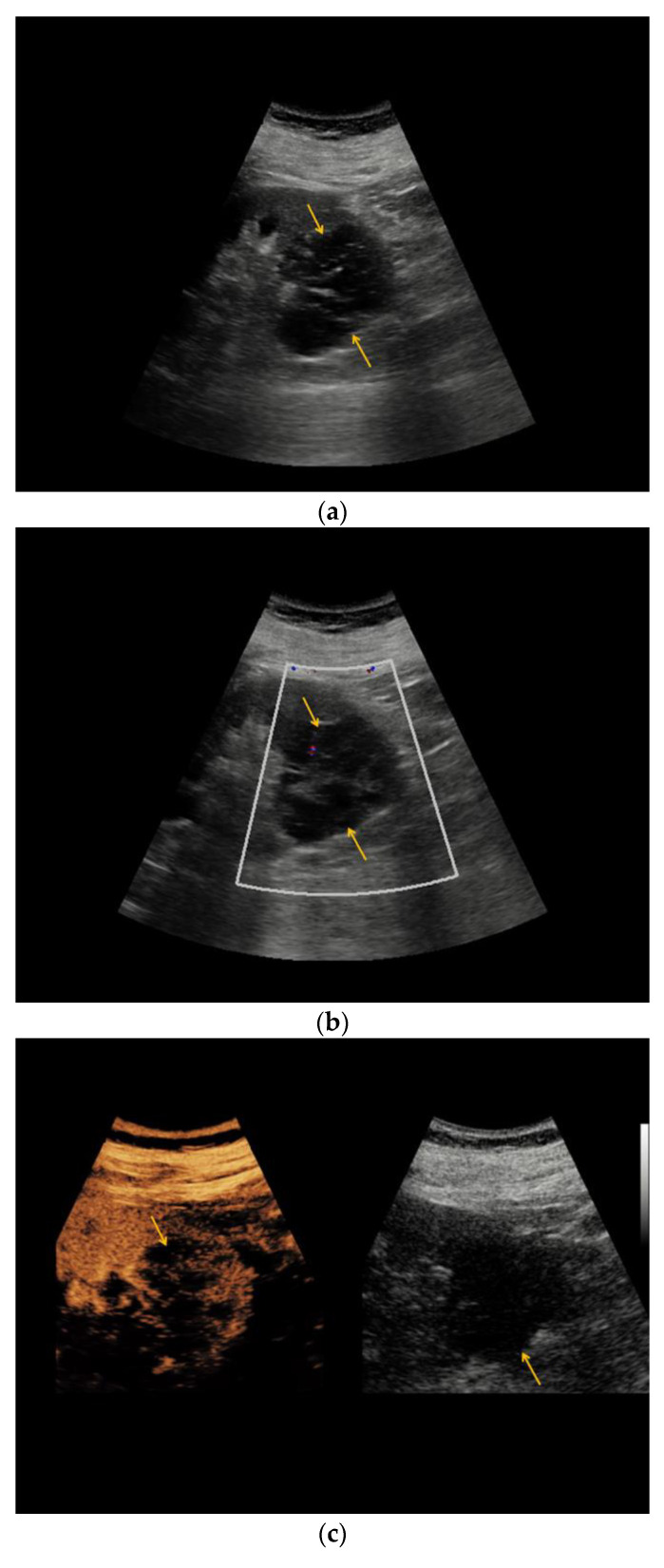

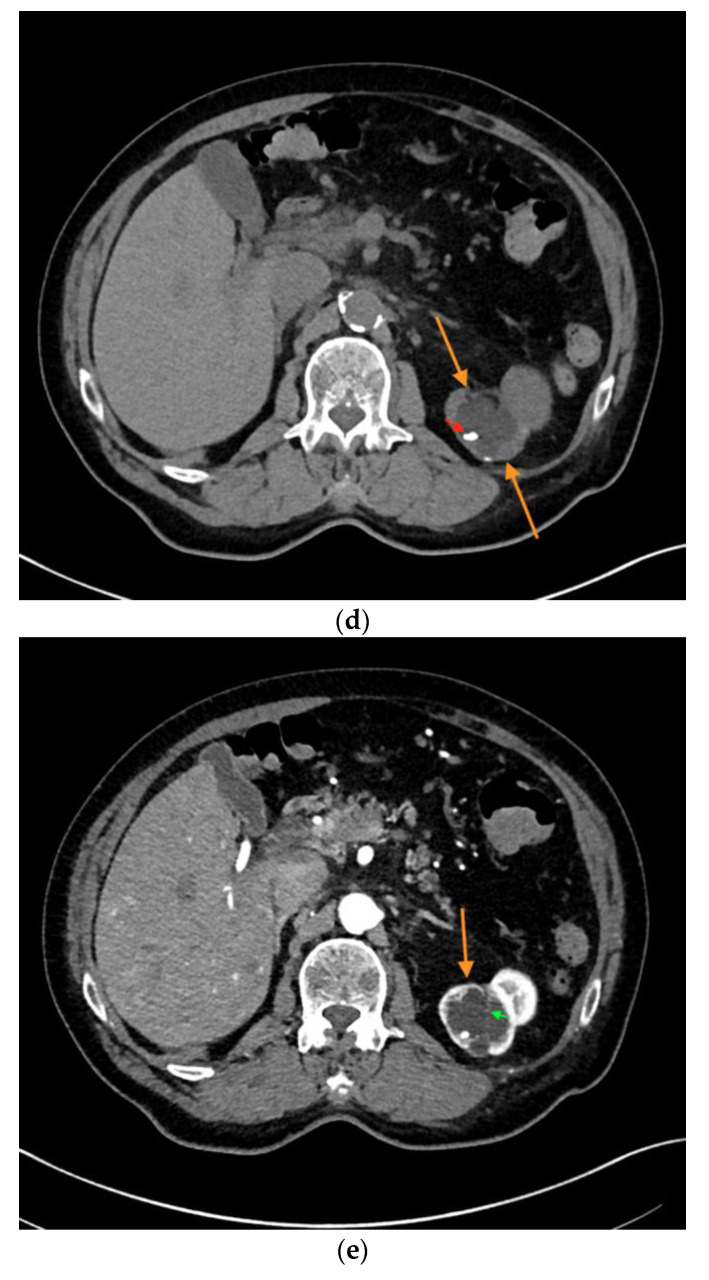
Complex renal cyst, Bosniak III subtype. (**a**) Inhomogeneous renal cystic lesion (orange arrows) with irregular septations is illustrated in native B-mode; (**b**) Neither perilesional nor intraseptal hypervascularization is detected in Color Doppler sonography; (**c**) Intraseptal contrast enhancement (orange arrow) is visualized by CEUS (left), displayed in a side-by-side manner with corresponding native B-mode (right). (**d**) Corresponding native computed tomography (CT) scan reveals focal mural calcifications of the renal lesion (small red arrow), axial reformation. (**e**) Discrete contrast enhancement of the septation is detected in contrast-enhanced CT (small green arrow), CT arterial phase, and axial reformation. The patient underwent nephrectomy, histopathology revealed clear-cell RCC.

In none of the included renal lesions, hypervascularization could be detected using Color Doppler ultrasound (Table 2). In 16/29 (55%) patients who underwent (partial) nephrectomy peripheral contrast enhancement could be visualized in CEUS, of which 10/16 (63%) and 6/16 (37%) histopathogically revealed to be malignant and benign, respectively. In 18/29 (62%) Bosniak III renal lesions, intraseptal contrast enhancement by CEUS could be detected, of which 11/18 (61%) and 7/18 (38%) turned out to be malignant and benign, respectively. In total, 4/29 (14%) renal lesions featured focally thickened septa which featured contrast enhancement, of which 3/4 (75%) turned out to be malignant. 6/29 (21%) renal lesions showed wash-out during late phase, half of which revealed to be the renal lesions turned out to be malignant. A representative appearance of a Bosniak III renal lesion is illustrated in Figure 2.

**Figure 3:**
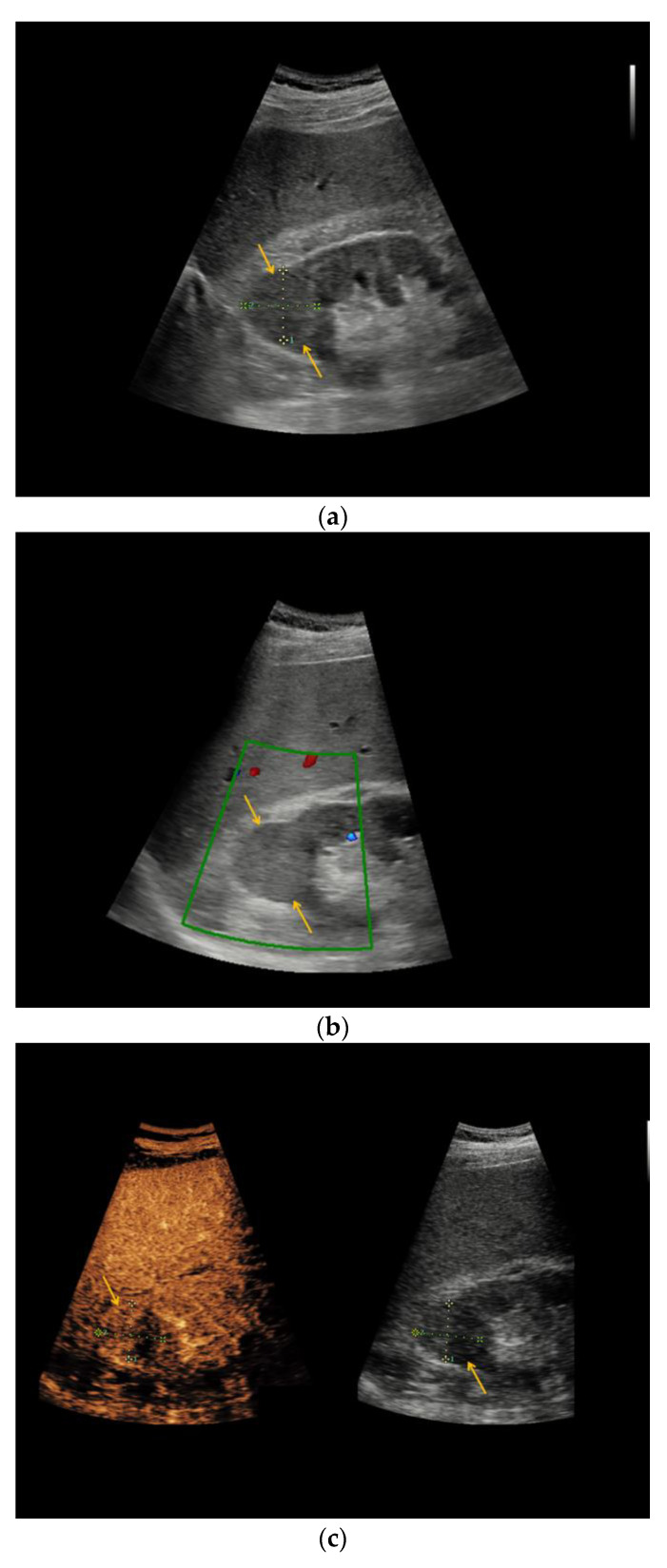

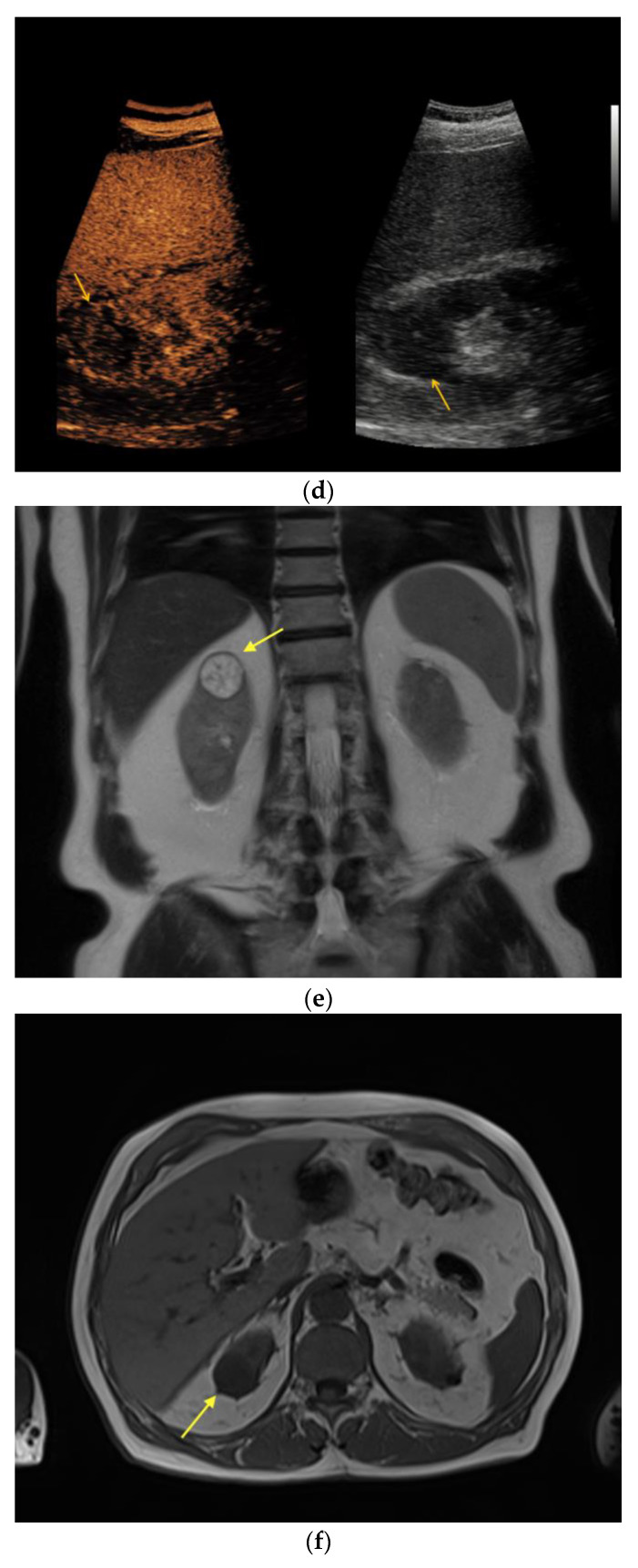

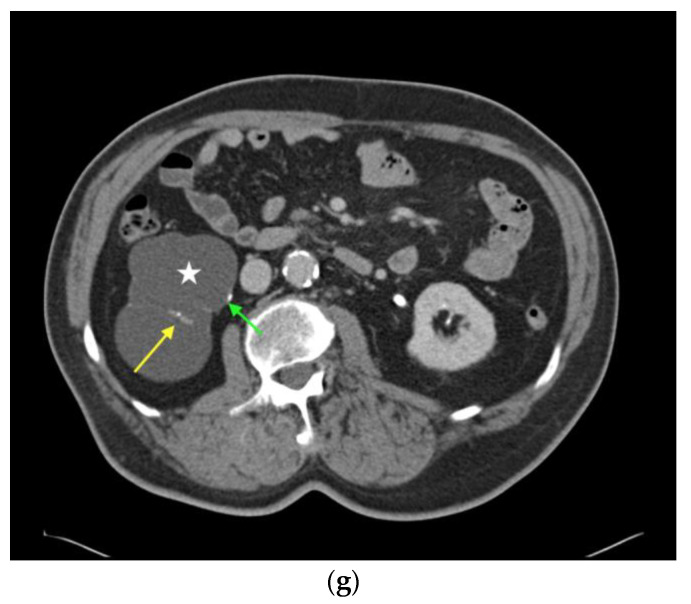
Renal oncocytoma in CEUS and CT. (**a**) In patient #16 inhomogeneous, round renal lesion in the upper pole of the right kidney is visualized in native B-mode (orange arrows). (**b**) No hypervascularization can be registered by Color Doppler sonography (orange arrows). (**c**) Early marginal contrast enhancement of the lesion is detected in CEUS (orange arrow, left), displayed in a side-by-side-manner with native B-mode (right). (**d**) Following delayed marginal wash-out is detected by CEUS (orange arrow, left), displayed in a side-by-side-manner with native B-mode (right). (**e**) Predominantly T2w-hyperintense correlate (yellow arrow) with T2w-hypointense septations in native magnetic resonance imaging (MRI), coronal reformation. (**f**) Corresponding T1w-hypointense appearance of the lesion (yellow arrow), native MRI, axial reformation. (**g**) In patient #27, a large renal cystic lesion (asterisk) with irregular septations (yellow arrow) and calcifications (green arrow) of the right kidney is shown by CT, venous phase, axial reformation. The patient underwent partial nephrectomy, renal oncocytoma was histopathologically confirmed.

In patient #1 additional MRI categorized renal cystic lesion as Bosniak IIF type, histopathology finally revealed clear-cell RCC. In patients #27 and #38, renal cystic lesions were categorized as Bosniak IIF by CT; histopathology revealed oncocytoma and clear-cell RCC, respectively. Figure 3 illustrates the heterogeneous morphology of two renal oncocytoma. Similar to the findings in CEUS, the renal cystic lesion in patient #14 was categorized as Bosniak III by CT, underlying cystic clear-cell RCC was histopathologically elucidated.

The remaining 20/49 did not undergo any urological treatment in our University Hospital, so final histopathological analysis is lacking. In patients #33, #37, #44 and #46, whereas CT or MRI described a hemorrhagic renal cyst, dynamic visualization of microperfusion by CEUS could show peripheral contrast enhancement of the cystic lesion. In patient #44, additional peripheral wash-out of the renal cystic lesion could be registered during late phase in CEUS and intraseptal contrast enhancement could be detected by CEUS in patient #46. Sonomorphologic features and thus Bosniak subtypes from CEUS examination of renal cystic lesions in patients #36, #40 and #48 matched the Bosniak categorization by either CT or MRI.

## 4. Discussion

The high diagnostic accuracy of CEUS to differentiate between malignant and benign renal lesions had previously been reported [14,18]. It could be shown that CEUS is more sensitive to contrast enhancement of renal cystic lesions than CT and MRI [19,20]. Due to its higher spatial and temporal resolutions, CEUS was reported to be superior to CT to detect contrast enhancement of tiny cyst walls, septa and solid parts of complicated cysts, thereby allowing for up- or downgrading of renal cysts according to the Bosniak classification system [21,22,23]. The aim of the present study was not to compare the diagnostic performance of CEUS versus CT/MRI, however two renal lesions were upgraded from IIF to III by CEUS, in both of which clear-cell carcinoma was revealed by histopathology.

CEUS showed equivalent diagnostic validity compared to more elaborate CT and MRI [24] in terms of assessing indeterminate renal lesions. Nevertheless, several studies described that CEUS like any other imaging modality is unfeasible to safely distinguish between RCC subtypes solely relying on qualitative imaging features. Due to overlapping morphologic features, some benign entities, benign complicated cysts or oncocytoma, may even be misinterpreted as malignant lesions by diagnostic imaging [25]. Although contrast enhancement is a typical feature of malignant lesions, 10/29 Bosniak III renal lesions which featured contrast enhancement, revealed to be benign by histopathology in our study. In our present study, three oncocytomas were categorized as Bosniak III renal lesions (patients #16, #21, #27) by CEUS, including one upgrading from Bosniak IIF to III (patient #27), thus prompting (partial) nephrectomy. Oncocytomas are described as predominantly benign tumors, only few case reports of metastasizing and infiltrative growth of oncocytomas are published. Thus, its benign entity still is debatable [26]. The sonomorphological overlap between renal oncocytoma and renal cell carcinoma was previously demonstrated [27,28,29]. So far, no sonomorphological feature has been established allowing for valid differentiation between oncocytoma and renal cell carcinoma.

Besides confirmed benign adult cystic nephroma, cystic hamartoma and papillary renal adenoma, hemorrhagic renal cysts were among the benign renal lesions which were categorized as Bosniak III.

Our findings demonstrate a malignancy rate of approximately 66% of CEUS-categorized Bosniak III renal cystic lesions. This percentage is relatively high comparing the striking heterogeneity of results from other studies. This might be explained due to the pre-selected study cohort at our Interdisciplinary Center at a University Hospital, the higher diagnostic accuracy of CEUS compared to mostly used and less accurate CT and MRI for stratification of renal lesions and the high experience level at which CEUS examinations were performed [30].

The cost-effectiveness of CEUS in comparison with CT and MRI in several abdominal diseases had already been described [31]. A recent work demonstrated the cost-effectiveness of CEUS over MRI to investigate incidentally found renal lesions. Their results showed less expensive diagnostic management of cystic renal lesions by CEUS compared to MRI. Accurate stratification of renal cystic lesions respecting the Bosniak classification is pivotal for subsequent clinical management of the patients. Besides from affecting patients’ health, unnecessary diagnostics and misdiagnosis may prompt inadequate treatment, thus resulting in maldistribution of financial resources.

The United States Food and Drug Administration (FDA) approved the use of CEUS in 2016 for liver applications, CEUS has since then obtained widespread acceptance for evaluating a broad range of different conditions [32,33,34]. A relevant cohort of patients in whom incidentally found focal renal lesions are detected often have comorbidities, including impairment of renal function, thyroid gland disbalances, allergic reactions or cardiac affections, thus depending on metallic medical devices like cardiac pacemakers. Hence, thorough evaluation before CT or MRI are performed is critical. With its excellent safety profile and less frequent adverse effects, CEUS may be performed in those patients with less hesitations and allowing for visualizing renal cystic lesions and possible microperfusion at higher spatial and temporal resolutions compared to CT and MRI. In case indeterminate renal lesions are detected in CT using inappropriate protocols, further ionizing CT scan can be avoided by using CEUS instead. In addition, advantages of non-ionizing CEUS are its wide accessibility and the possibility to directly repeat examinations at less frequent risks/complications than CT and MRI. CT and MRI are more expensive than CEUS and especially MRI is considered time-consuming. Moreover, CEUS allows for visualization of microperfusion in real-time at high frame rates for several minutes at multiple angles, thereby avoiding timing problems of image acquisition upon application of contrast agents. Compared to contrast agents used in CT and MRI, contrast agents for CEUS are purely intravascular contrast agents that do not diffuse into the tissue. In order to capture detectable contrast enhancement of renal cystic lesions by CT or MRI relevant concentrations of intravenous contrast agents are necessary. In critically ill patients, applying high volumes of contrast media may affect renal function and result in cardiac decompensation. In contrast, a comparably insignificant volume of contrast agents is required for CEUS overcoming those risks. Furthermore, in case renal lesions are incidentally found during ultrasound examination of the abdomen, CEUS can be additionally performed allowing for scrutiny and avoiding time delay which in turn would otherwise increase anxiety of the patient.

Up to date, recent clinical urological guidelines do not recommend CEUS as the primary imaging modality to analyze cystic renal lesions, but state it as an adjunct instrument [35]. Furthermore, CEUS is not included as an imaging modality in the recent Bosniak classification system [7]. The application of CEUS for accurately stratifying renal lesions according to the Bosniak classification system was already demonstrated in several studies [12]. Contrast-enhanced ultrasound proved to be more reliable in assessing complex renal lesions than conventional ultrasound [36]. Furthermore, CEUS showed equivalent diagnostic performance in evaluating complex renal lesions in comparison with CT and MRI [14,15,16,17,37]. The European Federation of Societies for Ultrasound in Medicine and Biology (EFSUMB) recommends comprehensive CEUS when indeterminate renal masses are incidentally detected in CTs, most of which are performed without an appropriate protocol for the evaluation of renal lesions [38]. Recently, the beneficial and promising role of CEUS for follow-up of Bosniak 2F lesions was demonstrated [15]. By using CEUS as a diagnostic instrument for follow-up, contrast-enhanced CT and MRI and their associated risks may be reduced. Moreover, by means of fusion imaging previously acquired data from CT and MRI scans may be integrated and processed in up-to-date ultrasound devices, allowing for real-time computerized fusion of cross-sectional images with ultrasound images in a real-time manner. Fusion imaging may allow to further evaluate focal renal lesions, particularly of previously as indeterminate described lesions [39].

Complementary to other recent studies, our data indicate that CEUS depicts a reliable imaging tool to scrutiny Bosniak III lesions [36]. Respecting the above-described assets of CEUS over CT and MRI- including diagnostic accuracy, safety profile and economic perspective- and general shortcomings of diagnostic imaging for evaluating renal lesions, CEUS should be considered a primary imaging modality for the assessment, appreciation the nature and probability of malignancy and guiding subsequent clinical management of renal lesions.

The study has several limitations. All patients were retrospectively included at one University hospital. All examinations were performed by one single radiologist using different up-to-date ultrasound systems.

To our knowledge, our study contains the largest cohort of CEUS-categorized Bosniak III renal lesions which were validated by histopathology.

Our findings are in line with previous studies and imply a promising role of CEUS in the diagnostic workup and precise stratification of renal cystic lesions, thereby guiding adequate clinical management in the future.

## Figures and Tables

**Table 1:** Malignant and benign histopathologic subtypes of Bosniak III type renal lesions categorized by contrast-enhanced ultrasound (CEUS). Percentages may not add up to due to rounding. RCC—renal cell carcinoma.

**Malignant (*n* = 19)**	***n***	**%**
Clear-cell RCC	12	41
Chromophobe RCC	1	3
Papillary RCC	3	10
Multilocular cystic RCC	1	3
Mixed RCC	2	7
**Benign (*n* = 10)**		
Benign epithelial (hemorrhagic) cyst	4	14
Oncocytoma	3	10
Papillary renal adenoma	1	3
Cystic hamartoma	1	3
Adult cystic nephroma	1	3

**Table 2:** Overview of clinical characteristics, imaging findings, treatment and histopathology of 29 patients with CEUS-categorized Bosniak III lesions. RCC—renal cell carcinoma, HU—Hounsfield units, L—left, R—right. CD—Color Doppler, CEUS—contrast-enhanced ultrasound, CT—computed tomography, MRI—magnetic resonance imaging, F—female, M—male.

Patient	Sex	Age	Location	Size (cm)	Native B-Mode	Vascularization (CD)	CEUS	CT	MRI	Treatment: Histopathology
#1	F	49	R	5.6	Cystic, septated	-	Intraseptal	-	Intraseptal enhancing → Bosniak IIF	Partial nephrectomy: Clear-cell RCC
#2	M	54	L	3.0	Cystic, hypoechoic areas, wall thickening	-	Peripheral	-	-	Partial nephrectomy: chromophobe RCC
#3	F	61	R	3.5	Cystic, septated	-	Peripheral, intraseptal	-	-	Partial nephrectomy: clear-cell RCC
#4	F	43	R	1.6	Cystic	-	Peripheral	-	-	Partial nephrectomy: clear-cell RCC
#5	F	64	L	5.0	Cystic, focally thickened, septa	-	Intraseptal	-	-	Partial nephrectomy: clear-cell partial cystic RCC
#6	M	74	L	2.0	Cystic	-	Peripheral, intraseptal	-	-	Partial nephrectomy: clear-cell RCC
#7	F	66	L	7.0	Cystic, complex	-	Intraseptal	-	-	Partial nephrectomy: multilocular cystic RCC
#8	M	54	L	1.5	Cystic, wall thickening	-	Intraseptal	-	-	Partial nephrectomy: clear-cell RCC
#9	M	76	L	1.6	Cystic	-	Peripheral, wash-out	-	-	Partial nephrectomy: papillary RCC
#10	F	66	R	1.5	Cystic	-	Intraseptal	-	-	Partial nephrectomy: clear-cell RCC
#11	M	75	L	2.7	Cystic, focally thickened septa	-	Intraseptal	-	-	Partial nephrectomy: Papillary RCC
#12	M	52	L	1.5	Cystic, focally thickened septa	-	Intraseptal	-	-	Partial nephrectomy: clear-cell RCC
#13	M	68	L	1.5	Partially cystic	-	Peripheral	-	-	Partial nephrectomy: clear-cell RCC
#14	M	86	L	4.0	Cystic, septated	-	Intraseptal	Septated, calcified, early enhancement, wash-out in delayed phase → Bosniak III	-	Nephrectomy: cystic clear-cell RCC
#15	M	63	R	2.0	Cystic	-	Peripheral	-	-	Nephrectomy: clear-cell RCC
#16	F	62	L	2.5	Partially cystic	-	Peripheral, intraseptal wash-in/wash-out	-	Native MRI: T2w: mainly hyperintense with hypointense septations T1w: hypointense	Renal biopsy: oncocytoma
#17	F	64	R	3.5	Cystic	-	Intraseptal	-	-	Partial nephrectomy: cystic hamartoma
#18	M	67	R	3.0	Partially cystic, focally thickened septa	-	Intraseptal	-	-	Partial nephrectomy: cyst, no malignancy
#19	M	71	L	1.2	Cystic	-	Peripheral	-	-	Partial nephrectomy: cyst, no malignancy
#20	F	48	L	10.0	Cystic, septated	-	Intraseptal	-	-	Partial nephrectomy: adult cystic nephroma
#21	M	76	R	2.0	Cystic	-	Peripheral and Intraseptal wash-in/wash-out	-	-	Partial nephrectomy: oncocytoma
#22	M	46	R	0.8	Cystic	-	Peripheral wash-in/wash-out	-	-	Partial nephrectomy: papillary adenoma
#23	F	69	L	8.0	Cystic, septated	-	Intraseptal	-	-	Partial nephrectomy: cyst, no malignancy
#24	M	69	R	2.5	Cystic	-	Peripheral wash-in/wash-out	-	-	Partial nephrectomy: Papillary RCC
#26	M	66	R	5.0	Cystic	-	Peripheral	-	-	Partial nephrectomy: Hemorrhagic, xantho-granulomatous cyst, no malignancy
#27	M	67	R	7.0	Cystic, septated, partially calcified	-	Peripheral, intraseptal	Septated, partially calcified → Bosniak IIF	-	Partial nephrectomy: oncocytoma
#32	M	83	L	4.7	Cystic	-	Peripheral	-	-	Partial nephrectomy: clear-cell RCC
#35	M	48	R	2.5	Cystic, septated	-	Intraseptal wash-in/wash-out	-	-	Partial nephrectomy: clear-cell RCC
#38	M	60	R	1.2	Cystic, septated	-	Peripheral	Septated, contrast-enhanced → Bosniak type IIF	-	Partial nephrectomy: clear-cell RCC

“-“ – No / None or not available.

## Supplementary Materials

The following are available online at https://www.mdpi.com/1010-660X/56/12/692/s1, Table S1: Overview of clinical characteristics, imaging findings, treatment and histopathology of all included patients.
