# Supplementary material for: Contrast-Enhanced Ultrasound (CEUS) for the Evaluation of Bosniak III Complex Renal Cystic Lesions—A 10-Year Specialized European Single-Center Experience with Histopathological Validation
Source: Medicina (Kaunas). 2020 Dec 12;56(12):692. doi: 10.3390/medicina56120692 (PMC7763943; doi:10.3390/medicina56120692)
Supplement: Supplementary file 1 [file medicina-56-00692-s001.pdf]

Table S1. Overview of clinical characteristics, imaging findings, treatment and histopathology of all included patients. RCC—renal cell carcinoma, HU—Hounsfield units, L—left, R—right. CEUS—contrast-enhanced ultrasound, CT—computed tomography, FU—follow-up, MRI—magnetic resonance imaging, F—female, M—male.

Patient	Sex	Age	Location	Size (cm)	Native B-mode	Vascularization (Color Doppler)	CEUS	CT	MRI	Treatment: Histopathology	Follow-Up
#1	F	49	R	5.6	Cystic, septated	-	Intraseptal	-	Intraseptal enhancing → Bosniak IIF	Partial nephrectomy: Clear-cell RCC	-
#2	M	54	L	3.0	Cystic, hypoechoic areas, wall thickening	-	Peripheral	-	-	Partial nephrectomy: chromophobe RCC	-
#3	F	61	R	3.5	Cystic, septated	-	Peripheral, intraseptal	-	-	Partial nephrectomy: clear-cell RCC	-
#4	F	43	R	1.6	Cystic	-	Peripheral	-	-	Partial nephrectomy: clear-cell RCC	-
#5	F	64	L	5.0	Cystic, focally thickened septa	-	Intraseptal	-	-	Partial nephrectomy: clear-cell partial cystic RCC	-
#6	M	74	L	2.0	Cystic	-	Peripheral, intraseptal	-	-	Partial nephrectomy: clear-cell RCC	-
#7	F	66	L	7.0	Cystic, complex	-	Intraseptal	-	-	Partial nephrectomy: multilocular cystic RCC	-
#8	M	54	L	1.5	Cystic, wall thickening	-	Intraseptal	-	-	Partial nephrectomy: clear-cell RCC	-
#9	M	76	L	1.6	Cystic	-	Peripheral, wash-out	-	-	Partial nephrectomy: papillary RCC	-
#10	F	66	R	1.5	Cystic	-	Intraseptal	-	-	Partial nephrectomy: clear-cell RCC	-
#11	M	75	L	2.7	Cystic, focally thickened septa	-	Intraseptal	-	-	Partial nephrectomy: Papillary RCC	-
#12	M	52	L	1.5	Cystic, focally thickened septa	-	Intraseptal	-	-	Partial nephrectomy: clear-cell RCC	-
#13	M	68	L	1.5	Partially cystic	-	Peripheral	-	-	Partial nephrectomy: clear-cell RCC	-
#14	M	86	L	4.0	Cystic, septated	-	Intraseptal	Septated,calc-ified, early enhancement, wash-out in delayed phase →Bosniak III	-	Nephrectomy: cystic clear-cell RCC	-

#15	M	63	R	2.0	Cystic	-	Peripheral	-	-	Nephrectomy: clear-cell RCC	-
#16	F	62	L	2.5	Partially cystic	-	Peripheral, intraseptal wash-in/wash-out	-	-	Renal biopsy: oncocytoma	-
#17	F	64	R	3.5	Cystic	-	Intraseptal	-	-	Partial nephrectomy: cystic hamartoma	-
#18	M	67	R	3.0	Partially cystic, focally thickened septa	-	Wash-in	-	-	Partial nephrectomy: cyst, no malignancy	-
#19	M	71	L	1.2	Cystic	-	Peripheral	-	-	Partial nephrectomy: cyst, no malignancy	-
#20	F	48	L	10.0	Cystic, septated	-	Intraseptal	-	-	Partial nephrectomy: adult cystic nephroma	-
#21	M	76	R	2.0	Cystic	-	Peripheral and Intraseptal wash-in/wash-out	-	-	Partial nephrectomy: oncocytoma	-
#22	M	46	R	0.8	Cystic	-	Peripheral wash-in/wash-out	-	-	Partial nephrectomy: papillary adenoma	-
#23	F	69	L	8.0	Cystic, septated	-	Intraseptal	-	-	Partial nephrectomy: cyst, no malignancy	-
#24	M	69	R	2.5	Cystic	-	Peripheral wash-in/wash-out	-	-	Partial nephrectomy: Papillary RCC	-
#25	F	35	L	12.0	Cystic, septated	-	Intraseptal	-	-	-	-
#26	M	66	R	5.0	Cystic	-	Peripheral	-	-	Partial nephrectomy: Hemorrhagic, xanthogranulomatous cyst, no malignancy	-
#27	M	67	R	7.0	Cystic, septated, partially calcified	-	Peripheral, intraseptal	Septated, partially calcified → Bosniak IIF	-	Partial nephrectomy: oncocytoma	-
#28	M	57	L	3.5	Cystic, septated	-	Peripheral, intraseptal	-	-	-	-
#29	F	75	L	1.0	Cystic	-	Peripheral	-		-	-
#30	M	78	L	4.0	Cystic, septated	-	Peripheral, intraseptal	-	-	-	-
#31	M	77	R	2.0	Cystic, septated	-	Intraseptal	-	-	-	-
#32	M	83	L	4.7	Cystic	-	Peripheral	-	-	Partial nephrectomy:	-

										clear-cell RCC	
#33	M	76	L	1.0	Cystic, septated	-	Peripheral	No contrast-enhancement, septated, calcified → hemorrhagic cyst, Bosniak II	-	-	CEUS (8 months, 30 months, 42 months): idem
#34	M	71	R	1.6	Partially cystic	-	Peripheral	-	-	-	-
#35	M	48	R	2.5	Cystic, septated	-	Intraseptal wash-in/wash-out	-	-	Partial nephrectomy: clear-cell RCC	-
#36	F	82	R	6.0	Partially cystic	-	Peripheral, intraseptal	-	Diffusion-restricted, peripheral T1-hyperintense, central T1-hypointense, central T2-hyperintense → Bosniak III	-	-
#37	M	76	R	2.0	Cystic, septated	-	Peripheral	Septated, inhomogeneous → hemorrhagic cyst, Bosniak II	-	-	-
#38	M	60	R	1,2	Cystic, septated	-	Peripheral	Septated, contrast-enhanced → Bosniak type IIF	-	Partial nephrectomy: clear-cell RCC	-
#39	M	92	L	3.5	Cystic	-	Peripheral	-	-	-	-
#40	M	65	L	4.0	Cystic, septated	-	Intraseptal	Septated, contrast-enhanced → Bosniak type III	-	-	-
#41	M	48	R	1.7	Cystic	-	Peripheral	-	-	-	CEUS (6 months, 12 months): idem
#42	M	41	R	6.5	Cystic, partially calcified	-	Peripheral, intraseptal	-	-	-	-
#43	F	82	R	1.5	Partially cystic	-	Peripheral	wall thickening, contrast-enhanced	-	-	-

								→ Bosniak type III			
#44	M	52	L	1.5	Cystic	-	Peripheral wash-in/wash-out	Hypernse (HU 40) → hemorrhagic cyst, Bosniak II	-	-	-
#45	M	77	R	5.0	Cystic, septated	-	Intraseptal	-	-	-	-
#46	M	49	R	3.0	Cystic, septated	-	Peripheral, intraseptal	-	Septated T2-hypotense Septa, no contrast-enhancement, no diffusion restriction → hemorrhagic cyst, Bosniak II		
#47	M	63	L	4.0	Cystic, septated	-	Intraseptal	-	-	-	-
#48	F	47	L	1.5	Cystic, focally thickened septa	-	Centripetal		Contrast-enhancing, mural thickening, → Bosniak III	-	-

“-“ – No / None or not available.
